# **“**Not yet a doctor”: medical student learning experiences and development of professional identity

**DOI:** 10.1186/s12909-022-03209-w

**Published:** 2022-03-04

**Authors:** Gyu Mi Park, Ah Jeong Hong

**Affiliations:** Department of Education, Chung-Ang University, Seoul, 06974 Republic of Korea

**Keywords:** Professional identity, Learning experiences, Medical students

## Abstract

**Background:**

To become a good doctor, developing a professional identity is as important as having the right knowledge and skills. Great attention has been given to professional identity in medical school because it plays an important role in the transition from student to doctor. Nonetheless, the necessity of acquiring a tremendous amount of knowledge and skill during medical school training does not create sufficient opportunities for students to develop their professional identities. Thus, this paper pays careful attention to how students’ learning experiences in medical school affect this development. The research questions are as follows. 1) How do medical students’ perceptions of doctors change or strengthen after entering medical school in the process of professional identity development? 2) What kinds of knowledge have medical students accumulated while attending medical school? How do their learning experiences affect professional identity development? 3) What is a doctor’s role and the career’s meaning to medical students, and what understanding does this awareness bring to their learning experiences and lives as future doctors?

**Methods:**

In-depth semi-structured interviews were conducted with 20 Korean students in their fourth year of medical school; all had more than one year of experience in clinical settings. The students’ learning experiences and professional identity development were used to analyze the data using inductive thematic analysis.

**Results:**

When students first entered medical school, they perceived their identities as “given to” them by society. However, various learning experiences during the medical school years affected them, causing them to think about becoming a doctor according to their own perceptions and the meaning of becoming a doctor in the profession. Although an isolated medical community and a competitive student culture hindered them from searching for their professional identities, informal learning experiences, including active interaction with patients, senior doctors, and others outside the medical community, enabled them to develop their professional identities. The medical students experienced a conflict between individual and professional values as they considered what kind of doctor they would be in the future.

**Conclusions:**

The findings noted in this study extend the understanding of professional identity and informal learning experiences in medical school.

## Background

A layperson becomes a professional doctor through completion of medical school. In medical education, developing a "professional identity" is essential to becoming a future doctor [1–3] in addition to medical knowledge and skills. Professional identity is “a representation of self, achieved in stages over time during which the medical profession's characteristics, values, and norms are internalized, resulting in an individual thinking, acting, and feeling like a physician” [4]. This identity enables students to gain confidence in what they are doing as doctors and affects their relationships with co-workers, professional groups, and patients [5].

However, medical students have not yet fully developed their professional identity as preliminary doctors. Most students enter medical school without an understanding of a doctor’s value [6]. Moreover, they have few opportunities to reflect on their professional identity after attending medical school [7]. As a result, many students have struggled to interact with patients and adopt the doctor role [8]. The lack of professional identity has returned to medical students as a negative impact on them [7–9].

One of the reasons that medical students cannot develop their professional identity can be found in medical education. Medical education has become a source of biased learning that excludes the humanistic aspects of medicine [10]. The way doctors' attitudes are reflected and reproduced in clinical practice has been ignored [11]. This is also related to the “dehumanization in medicine” problem caused by medical education, which mainly focuses on medical knowledge and technical skills [12–14]. Therefore, it is necessary to attract academic attention to medical education and professional identity development to make educational suggestions for creating better future doctors.

Earlier research has stated that medical students learn not only through the formal curriculum but through the informal learning of clinical practice to develop their professional identity [15]. Informal learning is “any activity involving the pursuit of understanding, knowledge or skill which occurs without the presence of externally imposed curricular criteria” [16]. Informal learning also occurs in a space surrounded by events and activities with a formal educational purpose [17]. For example, patient interaction in clinical practice [18–23], positive and negative role models [24–27], authentic experiences [28], and the medical community [29, 30] are aspects of informal learning that can affect professional identity. However, little attention has been paid to how students respond to informal learning in developing professional identity.

Adult learning is an individualized process that moves away from institutionalized learning and focuses on the meaning of individual learning and identity [31]. After entering school, adult learners reconstruct their identities in various learning experiences [32]. Thus, it is crucial to examine what unique learning experiences are accumulated in medical school and their meaning to medical students.

The research started with careful attention to how medical students develop their professional identity through learning experiences. Using the perspectives of informal learning and identity theory, this exploratory research focused on medical students' learning experiences within the social-cultural context of the Korean medical school’s learning environment and the meaning it provides to their professional identity development as future doctors. The research questions are as follows.

1. In the professional identity development process, how do medical students’ perceptions of doctors change or strengthen after entering medical school?
2. What kinds of knowledge have medical students accumulated while attending medical school? How do their learning experiences affect professional identity development?
3. What is a doctor’s role and the career’s meaning to medical students, and what understanding has this awareness brought to their learning experiences and lives as future doctors?

To this end, qualitative research was conducted with 20 Korean medical students in their fourth year. The study allows understanding of the medical students’ identity development and learning experiences, and it provides research implications, including suggestions for a better learning environment to educate medical professionals.

## Methods

Qualitative research is helpful to understand the in-depth meaning of individual experience [33]. This study was conducted to grasp the context of medical students’ learning experiences and deeply examine how these are involved in their professional identity development. To this end, the qualitative research method was adopted.

### Participants and setting

The participants consisted of 20 students in their fourth year at two Korean medical schools. A purposive sampling method was used to select suitable participants for the research purpose. Details on the setting and the criteria for selecting participants are as follows.

The Korean medical school program is six years long; the first two years are pre-medical courses that are followed by four years of clinical studies. The final one and a half years are usually based on clinical practice education. Korean medical students can become doctors if they pass the Korean Medical Licensing Examination (KMLE) national exam. After graduation, they can apply for an internship in a hospital. Their school records and KMLE scores determine the hospital where they can be an intern.

According to the Medical Act, Korean medical students can practice medical treatment such as history taking, physical examination, and clinical skills without a license. They must perform all medical activities under the guidance and supervision of their advisor, and they are responsible for performing medical treatment based on patient safety. Clinical practice is one of the essential educational strategies in medical school to develop medical students into primary clinicians with expertise and a sense of responsibility.

Considering the system of Korean medical education, it was most appropriate to select fourth-year undergraduate students with more than one year of experience in clinical settings as participants in this research. The researchers judged that they had had relatively enough time to think about the meaning of their learning experiences in medical school and had formed their professional identity through their career decisions.

The researchers contacted professors and doctors working in a university hospital to recruit the participants. Medical students who voluntarily agreed to participate in an interview and had been recommended by their professors and doctors were recruited. Finally, 20 fourth year students from two medical schools were selected; 14 were males, and 6 were females. Their ages ranged from the mid-20 s to the mid-30 s. They had completed most of their medical education course and were studying for the KMLE. The participants attended medical schools located in Seoul and Daegu city in Korea. Their schools’ names and other personal information were omitted to guarantee their anonymity.

### Data collection

To grasp the medical school curriculum in advance, the researcher referred to the medical school web page, which includes general information such as course types, teaching methods, credits, academic programs, and educational goals.

The data were collected through individual interviews. The participants were informed about the research purpose, content, and research ethics. The researcher and each participant had a conversation for about an hour in a classroom. All interviews were recorded with the consent of the participants. The recorded files were transcribed verbatim in Korean and translated into English later.

The interviews were conducted using semi-structured questions. A conceptual framework for the research questions was developed based on a literature review. The interview questions were based on the research questions. The key questions are as follows: “What motivated you to attend medical school?”, “What are the (dis)advantages of clinical practice in becoming a doctor?”, “What kind of (positive or negative) role models have you formed in your clinical practice?”, “What is the unique culture of the medical school?”, “What are the special perceptions of people toward doctors?”, “What would a “good doctor” look like?”, “When you compare yourself right after entering medical school with now, how has the image of the doctor you would like to become changed?” As the interviews progressed, the questions were elaborated on and enhanced to address the research questions better.

### Data analysis

In a qualitative study, data collection ends when it reaches data saturation in which new information cannot be obtained, and data collection cannot trigger new theoretical insights [34]. It has been recommended that between 16 and 24 interviews be conducted to reach a saturation of in-depth meaning in the responses to the research question [35]. A total of 20 interviews were conducted in this study when saturation was reached.

Data analysis was focused on medical students' learning experiences closely related to professional identity. This study used inductive thematic analysis [36] as a qualitative data coding and analysis method. Inductive thematic analysis is a flexible method for organizing and analyzing patterns in collected data. The analysis followed six steps: familiarizing with data, generating initial codes, searching for themes, reviewing themes, naming themes, and producing a report [36].

The researchers read the transcripts repeatedly to familiarize themself with the data and developed initial coding schemes extracting key ideas related to the research questions that emerged from the participants' interviews. Initial coding is the process of organizing the collected data, discovering meanings, and conceptualizing the results [34]. After that, the researchers categorized the data related to the themes: changes in the perception of doctors after entering medical school, common social perceptions of doctors and medical students, unique characteristics of the medical school community, informal learning (interaction, role models, authentic and a contextualized learning field) in clinical practice, and identity conflict as a prospective doctor were categorized. These were grouped into three overriding categories: job search and preparation, medical school learning experiences, and the meaning of a doctor’s profession. Each theme was named to clarify the analysis. Finally, vivid extracted examples were selected and produced to the research question.

In qualitative research, the trustworthiness of data must be considered [37]. The study used strategies to ensure trustworthiness. After explaining the study purpose to the participants, the interview was conducted, with the interviewer keeping a distance from categorizing the data. Two researchers reviewed the transcripts independently. They met weekly to cross-examine the categorization and interpretation of the data.

## Results

### Becoming a doctor: job search and preparation

The research participants who entered medical school with the dream of becoming a doctor began to explore the job by coming to understand a doctor’s life and the social perception of doctors in medical school over several years. This experience catalyzed the developing psychological readiness of ordinary students to become professional doctors while changing or strengthening their perceptions of doctors.

#### After entering medical school: from looking at to understanding a doctor’s life

The participants entered medical school for different reasons, such as their parent’s recommendation that they should become a doctor, the expectation of high job security, and people’s recognition that excellent students should attend medical school. The common theme in why they chose medical school is that they simply had followed others’ perceptions of a doctor.

*It goes without saying that the best students go to medical school. (Student 1, male)*

*When I was a high school student, I heard that doctors make a lot of money. I think I’ve been motivated by that. (Student 16, male)*

However, the participants changed their thinking after entering medical school and observing doctors' real lives. Before entering medical school, Student 11 thought the same as other people, "A doctor has a wonderful job respected by many people and earns a lot of money." But after clinical practice, she realized that a real doctor’s life was different from her earlier thought. A doctor’s role is never a job to earn lots of money and live comfortably. Student 6 also believed that a doctor could earn money easily. However, now she realized that being a doctor was not an easy job and that it takes much effort and time to become one.

*Doctors have to study continuously. They also must work in extreme situations with sick people. If you think being a doctor is a job to earn a lot of money and live comfortably, it's not such a job at all. (Student 11, female)*

*I didn’t know that it’s so hard. (…) I just knew that doctors earn lots of money and have a stable job, and they would be respected by other people. (…) But actually, it takes a lot of effort and time to become a doctor. It's a tough job. (Student 6, female)*

#### Social perception regarded medical students as “predetermined” doctors: a given identity

The participants were aware that the public gives special attention to medical students.

*When talking to my friends or relatives, I can feel that they envy me. Once, they said to me, “Our future doctor’s here.” (Student 11, female)*

There is a perception that doctors can make or break lives. This external perspective on doctors is being passed on to medical students.

*People usually think that doctors can make or break patients’ lives. So, it’s like; it makes me believe that human lives are in my hand. (Student 7, female)*

Student 6 said doctors and medical students have a sense of privilege because doctors are licensed and hold socially promising jobs. Those students who belong to the medical school community were identified as predetermined doctors.

*I think not only doctors but even medical students seem to have a sense of privilege. A doctor has a license, and it’s a socially promising job that has a bright future. (Student 6, female)*

### Learning experiences in medical school

From the perspective of informal learning, several characteristics of medical school learning experiences are closely related to professional identity development. The fiercely competitive, closed, and strongly authoritarian culture of the medical school community encourages passive learners who lack professional attitudes. On the other hand, the authentic learning environment of clinical practice gradually refines them into future doctors with professional competence, values, and attitudes.

#### Keen competition culture in medical school: a passive learning attitude

The participants describe that medical school looks quite similar to high school. The competition among students in Korean high schools with high “education fever” derived from the belief that entering a good university would guarantee one’s quality of life. A similar belief justified competition in medical school, assuming that choosing a popular specialty would guarantee a happy life. Korean medical students had to get better grades than others to choose such a specialty. In this competitive structure, the problem is that the issue of professional identity has been hidden by the student focus on picking a popular specialty because of their good grades. Instead of sufficient consideration of what kind of doctor they would be, they were chasing a popular specialty to occupy a better position than others and survive the competition.

*Eventually, a man who graduated from a famous university would have a good career and meet a pretty girlfriend. That’s the way things go. Now, I will have to graduate medical school with good grades to select the best specialty and then finish my internship here in the university hospital. After that, finally, I’ll become a doctor. The way of success in one’s life is quite similar. (Student 1, male)*

The participants were studying hard to get good grades. This means that there is no need to learn more than given tasks. In Student 3’s case, he would be willing to study hard only if it is necessary to graduate from medical school. The participants pointed to the “passive” learning attitudes of medical students.

*They are very passive, really. (Interviewer: Then, if it would be asked in the exam, would you?) Of course, I will study hard. If 20 credits for social participation are requirements for graduation, I will definitely do so. But if not, why do I need to do that? (Student 3, male)*

#### Medical students trapped in the cage of medical school

Doctors and students coexist in a medical school community. Doctors teach students, while students learn medicine from doctors. The relationship continues for a long time, even after students start working as qualified doctors. The study notes the characteristics of the medical school community and relationships with the community members to find how they influence students' identity development. One of the prominent characteristics of the Korean medical school community is that it is a “closed group.”

*It seems to be the problem of the medical group itself. It’s too closed. I think it may be worse than the army. (Student 3, male)*

The exclusiveness of the medical school community appeared as two types. The first was characterized by a lack of interaction among the community members. Some medical students with similar backgrounds formed an exclusive small group. Student 4 described that the tendency of such an exclusive small group was the expansion of a closed medical community, interrupting communication with patients and other doctors.

*There is a kind of culture that medical students usually hang out only with a group of friends who have similar backgrounds, making an invisible exclusive boundary around them. Many doctors and professors in medical school are exclusive, too. I think everything starts from those groups. Medical students become doctors who are not good at communicating with others outside of their group. (Student 4, male)*

The interaction between doctors and students in medical school is also restricted. According to participants who experienced a clear hierarchy in the community through clinical practice, there is a strong authoritarian culture.

*I thought if I do well, like providing good care for patients, everyone would like it. But what I’ve experienced in clinical practice was very different from my expectation. There’s a clear hierarchy among doctors and lots of constraints more than I thought. A hospital was another organizational society. (Student 1, male)*

The second type of closed medical school community is related to a lack of interaction with other academic groups. Medical school started with physical and psychological separation from non-medical groups due to the geographical location of medical school and the different school curricula. It caused indifference to people and society, restricting learning from interaction with various people.

*They should look for the outside world. Medical students have very narrowed and limited relationships. They don’t know anything other than medicine. (Student 8, female)*

*A doctor’s job is a stable one with a great social safety net. They don’t have to make efforts so as not to fall behind. Medical students are becoming complacent doctors, including me. I used to read many books and newspapers, finding out what happened these days in our society. But after four years have passed, now I don’t have any interest in that. (Student 3, male)*

#### Authentic learning

Clinical practice complements the limitations of lecture classes. The participants could understand the medical knowledge they had memorized in the class, observing diseases and symptoms. Some students regained interest in medicine and conducted self-directed learning through hands-on training.

*It’s no use just taking a class or looking at the picture of jaundice. Once you just look at the real patient, you will realize, “Oh, that's it.” No matter how hard I try to memorize the disease or accept it as an image, it can’t be better than observing real patients. (Student 3, male)*

Another advantage of clinical practice is that it can integrate medical knowledge learned only in a fragmentary way through a book. Beyond simply memorizing each disease along with its symptoms and treatment, students can identify the actual process by which medical knowledge applies to clinical practice. Student 5 said that while textbooks cover only one disease and treatment, priorities and treatment methods can be learned simultaneously when a patient has multiple diseases. These are not covered in textbooks.

*I’ve learned in a textbook about the treatment of a single disease, not the case of several diseases at the same time. In fact, the priority of treatment is not considered at all in the book. But in the case of clinical practice, it is helpful to understand the real treatment on how to care for patients who are suffering from many diseases at once. (Student 5, male)*

The experience of clinical practice was also helpful in specialty choice. Medical students were helped in their selection through contact with real patients and learning the workplace environment. Student 17 explained that students could choose their specialty based on their practice experiences considering their interest and aptitude.

*I think it’s the most important meaning of practice. Just as students are going to be different before and after entering medical school, practice also can change their minds about specialty choice. So, students may exclude some specialties that they didn’t really fit in with during practice, “I should not choose it. It does not fit me.” (Student 17, male)*

#### Finding a “good doctor” role model: A "friendly" doctor to patients

The key role models for medical students were the doctors they met in practice. Interestingly, the common characteristic of good role models was “kindness.” The participants chose doctors as good role models who stood out for their human side, not losing their kindness toward patients because of their busy schedules. Student 3 expressed his opinion that the doctor’s personality will emerge as an essential virtue in the future as much as the doctor’s competence.

*In the past, the doctor who knows every disease and treatment was judged as a competent doctor. But now, if you don’t know about something very well, then you can just look it up on the computer, smartphone, or something. All the information is showing up on the screen. So, I think it’s not really far away until the day of admitting the doctor who has a good personality rather than skills. If the doctor’s skill is 9 and the personality is 1, then it’s gonna be 7 to 3, or it’ll be possibly 6 to 4 in the future. (Student 3, male)*

The participants had not only positive but negative role models. Patient-friendly doctors were good role models. On the contrary, doctors who neglect patients and prescribe without making eye contact were negative role models.

*I’ve seen a professor who didn’t make eye contact with patients at all. The professor who lectured about how to treat patients in the hospital did it that way. Well, that is the gap between reality and ideals. (Student 4, male)*

#### Patients make good doctors

Clinical practice has become an essential learning space for medical students to develop identities through interaction with various people. The relationship with patients is critical to developing a medical student’s identity. The participants interacted directly with patients and formed values and attitudes as preliminary doctors. Student 13 notes that the perception of a specialty could change due to the relationship with patients.

*I was not interested in the specialty. But after the practice, I could feel lots of things by talking and communicating with patients so we could get to know each other. I changed my perspective on the specialty by myself. (Student 13, male)*

Meeting with patients greatly influenced identity formation. The participants interacted directly with patients and formed values and attitudes as preliminary doctors. Student 15 observed a patient with an incurable disease who could not accept it and missed a treatment period. Student 15 realized that a doctor could not cure every disease. Also, he learned that the competence to explain well to patients and help them accept their condition is necessary.

*I realized that doctors could not cure all illnesses. (…) I think if a doctor had explained better to the patient the disease the first time, the patient would have been able to accept rather than deny it for a long time. (…) At that moment, I felt that it’s important for doctors to cure patients, and it’s also important to help patients accept themselves by explanation. (Student 15, male)*

Medical students participating in practice have identities both as a “student” and a “doctor.” From the patient’s viewpoint, they are a doctor, while they are a student to doctors. These two identities conflict in the hospital. Patients were cooperating in treatment believing that medical students were real doctors. As medical students also knew about the belief, they were concealing who they were.

*If I don’t tell them (patients) I’m a student; they describe their symptoms very specifically. The people really trust me who believe that I’m a doctor. Then, it’s going to be very easy for me. But once I tell them the truth, they rarely come to talk to me and just say “yes” to all of my questions. (Student 14, female)*

On the other hand, medical students were viewed as students still training to become doctors by professors and senior doctors. They do not belong to the hospital, nor are they qualified as doctors. They think of themselves as students evaluated by doctors, referring to "their weakest position" in the hospital.

*We’re students in the weakest position who are always conscious of doctors and professors. I thought, “If I do something wrong, I can’t get good grades.” So, I couldn’t be active in practice. I was going to be uncomfortable with doctors and professors only because of their one word. (Student 13, male)*

The participants regarded themselves as students rather than preliminary doctors, avoiding their responsibilities to the patients in clinical practice. Student 8 added, as she was reflecting on herself, that she was indifferent to patients because they were not her patients.

*I tend not to examine patients’ conditions in detail. When I get feedback from my professor, I thought, “Did I miss it because they are not my patients?” (Student 8, female)*

The participants gave various responses to the question of “when will you admit you are a doctor?” The responses were, “after graduating from medical school, have opportunities to participate in treatment directly,” “when becoming one’s doctor and get some experience,” or “take on a doctor’s role and responsibility.” The common element in the responses is that the students think abundant clinical experience is necessary to admit they are a real doctor.

Interns are doctors. They have a name card. If I’m an intern, I will meet patients confidently. Interns can draw blood for testing and get consent from patients or something. But students can’t do those things. Actually, nothing could be done by students. (Student 14, female)

*I’ll do better when I become someone’s doctor. Interns just do a job with chores. They don’t have their own patients. So, I think it would be different after I have my own patients later. (Student 8, male)*

*I think the doctor’s role is different depending on the position. For example, as an intern, if you can draw blood and release patients with discomfort at night, I guess it’s enough to say I am in an intern’s position. So, what I want to say is that I should admit I am a doctor, like, "Oh, I think I am a doctor now.” (Student 20, male)*

### The meaning of being a doctor

While attending medical school, the participants developed their own awareness of the doctor’s role and the career’s meaning. The awareness clarified why they had to learn in a certain way in medical school and what values they must pursue as future doctors.

#### Becoming a doctor

Most of the participants expressed that they had difficulty studying. They had to memorize large amounts of medical knowledge and confirm it through tests. This type of learning environment is far from one that encourages them to be interested in medical study.

Doctors always looked cool to me. But now, I know it’s not easy to live as a doctor. I have too much work to do. Rote learning seems to make me lose any interest in studying. (Student 8, female)

Even though the participants had trouble adapting to medical classes focusing on memorizing a large amount of content, they regarded it as an inevitable strategic choice associated with becoming a doctor. Student 15 thought that medical knowledge is essential for doctors, so rote learning was the most effective way to absorb such knowledge in a short time. Therefore, the medical classes were neither interesting nor unsatisfying to him.

To get a lot of knowledge in a limited time, efficiency is the most important thing. I also think the most important thing for a doctor is knowledge, not creativity. I believe that I need to know what I need to know. So, I don’t have any fun along the way, but I don’t complain, either. (Student 15, male)

Realizing that there was not much time until they received a doctor's license, the participants worried about what they had learned in medical school and how much they knew about it. A doctor's duty to treat patients with accurate medical knowledge without any mistakes has come to mean fear and responsibility. The resolution to be faithful to the doctor’s duties became a driving force to study hard.

I have some worries about myself. For example, I didn’t know that before, but now that it’s about time to get a doctor’s license, I feel like I have a tremendous responsibility for my judgments. That makes me extremely nervous. (Student 7, female)

It’s most important to know medical knowledge accurately. (…) We are the ones who can do harm to others if we don’t know exactly. So, all of us think that it’s really important to know precisely and correctly. That’s why we have to study hard. (Student 6, female)

As for the specific atmosphere of the hospital and medical school, the participants pointed out some problems but deemed to some degree their necessity as well. As mentioned above, medical schools and hospitals are characterized by a strong hierarchy and authoritarian culture. The participants regarded that this culture was formed in an effort to keep health services safe.

The hospital itself is a place where we are treating human life, and it’s where we should be alert. (Student 13, male)

There is a distinct culture in medical school. Having attended another school, Student 6 talked about the special culture only seen in medical school. At the school from which she had graduated, idiosyncratic personalities were naturally accepted. In contrast, the medical school had a “standardized framework.” In medical school, exhibiting behaviors that stood out was regarded as “strange.” Student 15 thought that hiding their unique personality was related to the characteristics of doctors’ work. The act of emphasizing one’s personality, such as through creativity and challenging behavior, could threaten a patient’s life. It is a top priority for doctors to treat patients safely by precisely applying earlier treatment rather than engaging in challenges or creativity.

Doctors are not supposed to be challenging. I think it’s not right to reveal individual creativity in medical treatment. It can put patients at risk. (…) So I think the individual character should not be emphasized. So, the culture and atmosphere are quite understandable. (Student 15, male)

#### Conflict between individual value and professional value

There were two conditions for choosing a popular specialty among medical students. First, a field’s associated income level should be good. Second, its work intensity should not be high.

Popular specialties usually meet two conditions. The income should be good, and then the working hours should be short, which means that the work intensity is weak. (Student 15, male)

The participants' conflicts between personal and professional values increased their concerns about career choices. They were conflicted between a specialty in which it is easy to manage both work and household and the specialty in which they truly want to work. Student 7 was deep in thought about whether she should choose the specialty she wanted or another, which is considered to be good for quality of life. Her decision became more complicated after she heard a professor’s story. The professor chose to do minor surgery instead of major surgery to manage both work and childrearing.

The older I get, the more I want quality of life. One day the professor told me her story. She wanted to become a great surgeon in the past. But after she married and then gave birth to a child, she decided to do small surgeries. She said priorities are changed in a lifetime. (…) I hope to be a ○○ doctor if I could, but should I have to live so hard? (Student 7, female)

Student 20 considered high income a top priority in specialty choice, but his values changed as he participated in clinical practice. He could understand the meaning of chronic illness in a patient's life that the books never revealed. Now he considers a specialty that requires interaction with patients who may give meaning to his life as a doctor. He said, "I’m not old enough yet,” as the purpose of becoming a doctor is stepping away from money and trying to pursue something more than material value.

Money couldn’t be ignored. (…) Material is, of course, it’s good. But I guess there is something more than material. Maybe I’m not old enough yet. (Student 20, male)

## Discussion

When an ordinary student forms a professional identity as a preliminary doctor from entrance to graduation from medical school, it is a dynamic process. At first, the students who entered medical school followed the perceptions of others about doctors, and they simply admired doctors as people commonly do. In addition, they identified themselves as predetermined doctors, showing a sense of privilege, pride, and self-confidence. In Korea, education is regarded as the path of success to occupy a higher socio-economic status, demonstrating the effects of a Confucian cultural heritage [38]. The social perception of doctors revealed in the study also reflected the common belief from Confucian culture that top students will certainly enter medical school and be educated to become doctors who can live successful lives with high social status. They thus were absorbing an identity derived from the social perception of medical students as doctors, which means that success is a foregone conclusion.

Medical students’ professional identity shows their “possible selves,” which means the self-presentation of what they would like to become and what they are afraid of becoming [39, 40]. The doctor's identity reveals a possible self for medical students in clinical practice. This identity has a weaker social binding power than the current self. Meanwhile, the current self, the student identity, remains stable due to social feedback such as that associated with assimilation into the medical organizational culture. Multiple selves are activated in accordance with the social environment [39].

The situation changes as they continue their school life. They withdraw from the image of a doctor derived from social perception and start to think for themselves about what it is like to be a doctor. The given identity reflects the social expectation that a medical student can be a promising doctor who can treat every disease. The participants were confident and had adopted the given identity, but they were afraid of its expectations simultaneously. They clearly recognized the limitation that a doctor never cures all illnesses. The fact that they have to take care of patients has come with heavy responsibility.

The study shows that identity is formed dynamically by the intricate entanglement of individuals and society rather than in a linear or fixed way [1, 41] through the professional identity development of medical students. The identity of a medical student is not merely that of a doctor but that of a person who would be reborn as a doctor with congruent professional behaviors, attitudes, and values. The participants hoped to be a warm-hearted doctor like their role models who treat patients well. The way to become the doctor they hope to be depends on developing professional identity through their rich clinical experiences, passion, and effort. The medical students must become “agents” [42] who can lead their own professional identity formation.

The first step toward becoming an agent of identity is to embrace a doctor’s life as it is. The participants found out why they should keep learning by rote and why they should assimilate into the culture of the medical community. Rote learning was the best choice for them to acquire more knowledge of medicine in a short period to become a decent doctor. The medical community's authoritarian and strongly hierarchical culture was a necessary evil that protects patients.

The meaning of professional identity to medical students could be interpreted as taking on a “social identity” [43] which concentrates on social group attributes. The social identity is individual self-conception, derived from membership in a social group, and accompanied by the values and emotions fixed in membership [44]. Having a social identity means performing actions similar to other group members, employing the group’s perspective, and reinforcing the group's consistent values, perceptions, and behaviors [45]. The participants tend to frame their behaviors to mimic other medical students rather than emphasize their individuality. This shows "deindividuation," which refers to a contextual change of identity from an individual to a group member [46]. One unique individual, a medical student, acquires social identity in being labeled a professional group member.

In becoming a doctor, medical students are confronted with a new conflict. The conflict is the question of what value should they bring to the job, an individual value or a professional one? The struggle with the choice between work and family [47] represents a person who has undertaken various social roles with multiple identities in society [48]. The internal confusion of medical students in this conflict is related to psychology’s identity theory that links individual roles to identity [43]. Individuals with multiple identities negotiate their roles with identity role partners, manipulate the environment to control the resources responsible for the roles, and meet role expectations [45]. The study participants looked for their roles in two different social spaces, the hospital, and the household. In the hospital, they negotiated their roles with the role partners of patients and doctors. In their personal lives, they were concerned about their roles as heads of present or future families. The career choice accounting for their reality represents an attempt to manipulate the surrounding environment to fulfill the responsibilities accompanying both their work and individual lives.

It is suggested that medical education should facilitate professional identity formation so that students can discern robust and compelling values ​​as future doctors. As society has become more complex, the difficulties of harmonizing multiple identities have increased [49]. A robust professional identity will help promote mature career decision-making by assisting the integration of self and occupation [50]. Therefore, if educational support for the development of professional identity within the medical school learning environment is achieved, students’ identity confusion and value conflict would be reduced. As agents of identity, they would find what their job means in life.

This research reveals that medical students’ professional identity is affected by various types of learning experiences in medical school. The most striking feature of the Korean medical school community is its closed structure, marked by competition and an authoritarian culture that hinders interaction among people. In this environment, the participants had great difficulty communicating with others. It was promoting doctors who are indifferent to patients and society, preferring to pursue their own interests. The purpose of becoming a “good student,” not a “good doctor,” has overtaken the opportunity to grow as a social being.

Based on the results, it is necessary to have a learning environment that enables medical students to interact with people to develop a professional demeanor. This can be discussed within the category of informal learning. Informal learning is contextualized [51]. The greatest advantage of clinical practice is that students encounter patients and doctors in a context-based clinical environment. Some participants started to think about the meaning of diseases in patients’ lives and behaviors as a doctor through clinical immersion. Interaction with other doctors also provided meaningful learning. Medical students took as role models doctors good at interacting with patients; they then behaved in ways that reflected the role models’ behaviors. While living in a hospital with doctors, medical students learned a way of doing and a way of thinking. The rich learning space enabled them to use existing knowledge to deeper understand and develop personal and professional values. The study shows that improved doctor-patient interaction is needed to foster good doctors [52]. Therefore, efforts to provide an interactive learning environment should be attempted to understand patients and care for them according to the humanistic aspects ​​of medicine.

The study has some limitations. A small sample from two Korean medical schools was used in the research. Since the government controls the nationwide medical school curriculum in Korea, it is expected that there would be little regional variation in exploring students’ learning experiences.

It is worth examining or comparing professional identity development according to students’ unique informal learning experiences as adult learners using larger samples from Korea and other countries. For example, a study conducted in Brazil confirmed the impact of the hidden curriculum of the professional identity, a “speeding up” culture that requires quick work and pressure to learn as much as possible without sufficient reflection on clinical experience, which ends with a lack of awareness of students’ professional identity in the context of a chaotic health care system [53]. Another study, conducted in an Australian university, found that students from diverse cultural backgrounds had two main senses of their professional identity [54]. One is professional inclusivity which is formed when they are treated as future doctors. The other is social exclusivity which is developed by socially separating themselves from non-medical students [54]. Future research can be expected in international studies conducted in similar or different socio-cultural contexts from that of Korea.

## Conclusions

This study investigates medical students’ professional identity development through informal learning experiences in a distinctive Korean educational environment. The study indicates that medical students, in their earlier years, accept a “given identity” provided by social perception. The limited interaction in the medical school community and competition culture obstructs professional identity formation. On the other hand, informal learning in clinical practice, full of interaction, contexts, and authenticity, facilitates students’ thinking about what kind of doctor they would be as the agent of their identity. They experience a conflict between individual and professional values, in considering what kind of doctor they will be in the future. In the process, difficulties in medical school were accepted as preparation for becoming a doctor by medical students. The findings noted in this study extend the understanding of professional identity and learning experiences in medical school.

## Data Availability

The raw data supporting the conclusions of this article will be made available by the authors without undue reservation.

## Abbreviation

KMLE: Korean Medical Licensing Examination
